# Growth Characteristics of Ramet System in *Phyllostachys praecox* Forest under Mulch Management

**DOI:** 10.3390/plants13131761

**Published:** 2024-06-26

**Authors:** Guibin Gao, Xing Wen, Zhizhuang Wu, Hao Zhong, Yanhong Pan, Xiaoping Zhang

**Affiliations:** 1 China National Bamboo Research Center, Key Laboratory of State Forestry and Grassland Administration on Bamboo Forest Ecology and Resource Utilization, Hangzhou 310012, China; anshu998@caf.ac.cn (G.G.); wenxing202202@163.com (X.W.); wzzcaf@126.com (Z.W.); zhonghao0726@163.com (H.Z.); zhukan2004@163.com (Y.P.); 2 National Long-Term Observation and Research Station for Forest Ecosystem in Hangzhou-Jiaxing-Huzhou, Plain, Hangzhou 310012, China

**Keywords:** *Phyllostachys praecox* C.D. Chu et C.S. Chao ‘Prevernalis’, bamboo ramet system, rhizome morphology, bud bank, branching

## Abstract

The ramet system is a typical structural type in the life history of clonal plants. This massive structure is formed by many similar ramets connected by underground rhizomes, which are independent and mutually influential. Therefore, the ramet system is unique to bamboo forests, and its role in the construction, maintenance, and productivity of bamboo populations is irreplaceable. Mulch management is a high-level cultivation model for bamboo forests that is used to cultivate bamboo shoots. However, the basic conditions of bamboo ramet systems in this managed model are poorly understood. This study analyzed the underground rhizome morphology, bud bank, and branching of bamboo ramets in a *Phyllostachys praecox* C.D. Chu et C.S. Chao ‘Prevernalis’ forest to explore the growth patterns of bamboo ramets in high-level management fields. In mulched bamboo forests, the bamboo rhizomes, distributed in intermediate positions of the bamboo ramet system, were long with many lateral buds and branches, and those at the initial and distal ends were short with few lateral buds and branches. The initial end of the ramet system reduced the ramet system, the intermediate part expanded the ramet system, and the distal end promoted ramet system regeneration. Owing to the continuous reduction, expansion, and renewal of ramet systems, the bamboo rhizome system demonstrates mobility and adaptability. This study found that a higher level of bamboo forest management increased the possibility of artificial fragmentation of the ramet system and that improving the efficiency of the ramet system was beneficial for maintaining its high vitality. Thus, this study provides a crucial reference for guiding the precise regulation of bamboo ramet systems in artificial bamboo forests.

## 1. Introduction

Bamboo is an economically important plant in China [1], with bamboo shoots and timber being the main products. Bamboo shoots have been consumed in China since ancient times and offer significant economic benefits owing to their high price, large production volume, and high consumption. Because of the rapid growth, short production cycle, and high nutritional value of bamboo shoots, they are considered a sustainable nutritional resource for the present and future and a means of ensuring food security [2]. The widespread use of bamboo shoots has promoted the vigorous development of bamboo forest cultivation for bamboo shoots. The cultivation of bamboo forests for bamboo shoots uses an intensive management type with high inputs and outputs, whereby mulch management is critical and uses a high-level cultivation model [3,4]. China’s climate varies significantly from north to south. For many bamboo species distributed in northern China, during winter, the shoot buds nurtured by their underground rhizomes enter a dormant state due to low temperatures and germinate gradually when the temperature rises the following year. Bamboo farmers drew inspiration from off-season cultivation techniques of vegetables and fruits in agricultural production and mulch the surface of the bamboo forests with organic materials, such as straw and rice husks. They utilize the heat generated by microbial reproduction, decomposition, and fermentation of the organic coverings to break the dormancy period of underground shoot buds, promote shoot bud differentiation, and achieve the goal of spring shoots emerging in winter [5,6]. Notably, the bamboo shoots’ emergence from the mulched forests coincides with the Spring Festival, a traditional Chinese festival.

Clonal plants have strong adaptability to their growth environment [7]. Bamboo is a clonal plant with highly sensitive phenotypic plasticity to environmental changes [8,9]. The high-level intensive management model and meticulous cultivation of mulched bamboo forests have substantially affected their growth [10,11,12]. The most critical characteristic of asexual reproduction in clonal plants is the ability to produce many similar ramets that can be connected through underground rhizomes for a certain period, forming large structures that occupy a large area [13]. This ramet system (clonal fragment) is referred to as a structural type of clonal plant [14]. Moreover, bamboo mainly propagates asexually and can connect its various ramets through underground rhizomes to form a ramet system. This allows for resource sharing through clonal integration [15], generating corresponding ecological adaptation strategies for various survival conditions. As the main form in the bamboo life history, ramet systems play a crucial role in the construction and maintenance of bamboo populations. However, studies on this topic are limited.

The regeneration of bamboo forests is mainly achieved through the lateral bud branching of underground rhizomes. Clonal plant bud banks usually undergo changes owing to environmental and external disturbances [16]. We conducted preliminary research on the characteristics of bud populations in mulched bamboo forests [17] and found that mulching significantly affected the bamboo bud banks, causing high-intensity bud output phenomena. Furthermore, we conducted control experiments on potted bamboo seedlings in the soil of bamboo forests from different mulching management years [18], studying the reproductive process of bamboo from a single-mother bamboo to a ramet system. The results showed that bamboo forest mulching not only inhibited branching but also caused differential reactions of different branching types.

To provide useful insights, researchers must investigate the status of bamboo ramet systems in the field, the impact of artificial management on their growth, the relationship between ramet systems and productivity, ramet systems versus the environment, and ramet systems versus ramet systems. Therefore, we conducted a growth investigation on the bamboo ramet system in mulched bamboo forests, using the famous Chinese “vegetable bamboo”—*Phyllostachys praecox* C.D. Chu et C. S. Chao ‘Prevernalis’. We evaluated the underground rhizome morphology, bud banks, branching, and branch distribution patterns of the bamboo ramet system in the field. These results provide an important basis for the further implementation of the precise regulation of bamboo ramet systems in artificial bamboo forests.

## 2. Results

### 2.1. Underground Rhizome Morphology of Bamboo Ramet System

**Figure 1:**
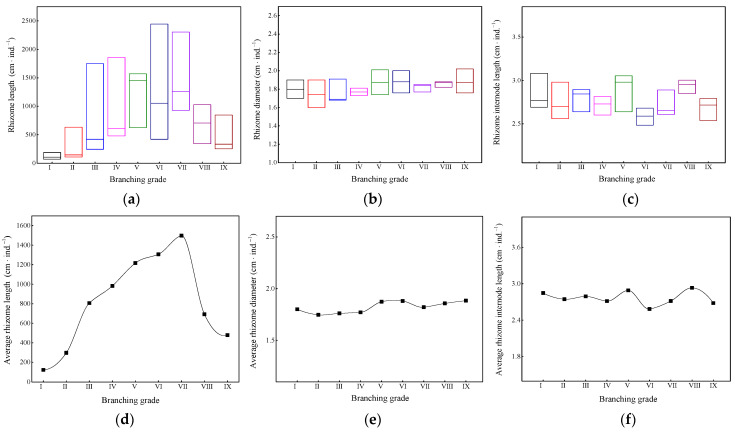
(**a**) Variation ranges of rhizome length; (**b**) rhizome diameter; (**c**) rhizome internode length; (**d**) variation trends in average rhizome length; (**e**) average rhizome diameter; (**f**) average rhizome internode length. Unit: cm·ind.^−1^ is cm per individual ramet system.

As shown in Figure 1, the length of the underground rhizome in branching grades III–VII of these bamboo ramet systems varied widely (Figure 1a). As the branching grade increased, the average rhizome length showed a trend of first increasing and then decreasing (Figure 1d). The average rhizome length of the branching grade VII reached 1497 cm (Figure 1d). The ranges of the variation in rhizome diameter (Figure 1b) and internode length (Figure 1c) at the same branching grade were relatively small. As the branching grade increased, the overall fluctuations in the average rhizome diameter (Figure 1e) and average internode length (Figure 1f) were not significant. Thus, the bamboo ramet system existed in a morphological structure of “large in the middle and small at both ends”. During the expansion of the ramet system, there were significant differences in the length of the bamboo rhizomes, and the differences in rhizome diameter and internode length were relatively small.

### 2.2. Underground Bud Bank of Bamboo Ramet System

**Figure 2:**
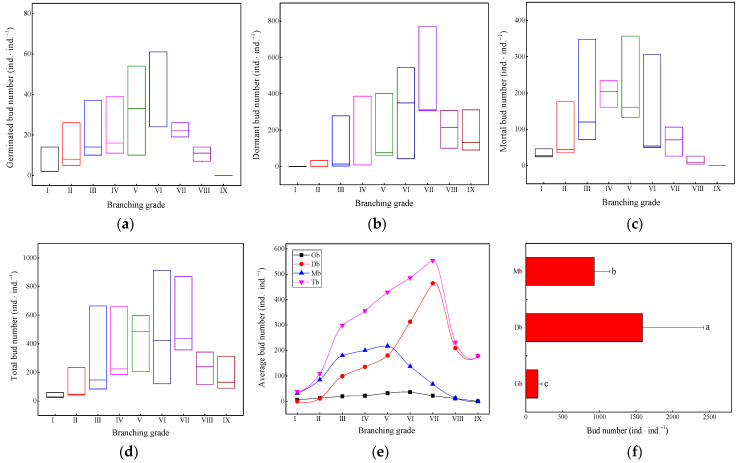
Quantitative changes in bud bank structure. (**a**) Germinated bud number; (**b**) dormant bud number; (**c**) mortal bud number; (**d**) total bud number; (**e**) average bud number; (**f**) differences in bud quantity. Mb: mortal bud, Db: dormant bud, Gb: germinated bud. Different letters indicate significant differences (*p* < 0.05). Unit: ind.·ind.^−1^ is individual bud per individual ramet system.

As shown in Figure 2, the ranges of the changes in the germinated buds (Figure 2a), dormant buds (Figure 2b), mortal buds (Figure 2c), and total number of buds (Figure 2d) in the intermediate branching grade of the ramet system were relatively large. Branching grade IX of the ramet system had mainly dormant buds (Figure 2b) but neither germinated buds (Figure 2a) nor mortal buds (Figure 2c). Branching grade I mainly had germinated buds (Figure 2a) and mortal buds (Figure 2c) but not dormant buds (Figure 2b). The average number of lateral buds at each branching grade showed a single peak (Figure 2e). As the branching grade increased, the changes in the average total number of buds and the number of dormant buds were significantly greater than those of mortal buds and germinated buds, achieving a significant increase from branching grade III and reaching their peak at branching grade VII. The average number of mortal buds was higher than that of dormant and germinated buds at branching grades I–V and peaked at branching grade V. The average number of germinated buds peaked at branching grade VI. The average total number of buds in the underground rhizomes of the ramet system was 2682 ind.·ind.^−1^, with the highest number of dormant buds exceeding 1500 ind.·ind.^−1^; there were many mortal buds close to 1000 ind.·ind.^−1^; and the minimum number of germinated buds was less than 200 ind.·ind.^−1^ (Figure 2f). These results indicated that the metabolic and growth costs of lateral buds germinating into branches in bamboo ramet systems were high, and the cost of dormant buds was relatively low.

### 2.3. Branching of Bamboo Ramet System

**Figure 3:**
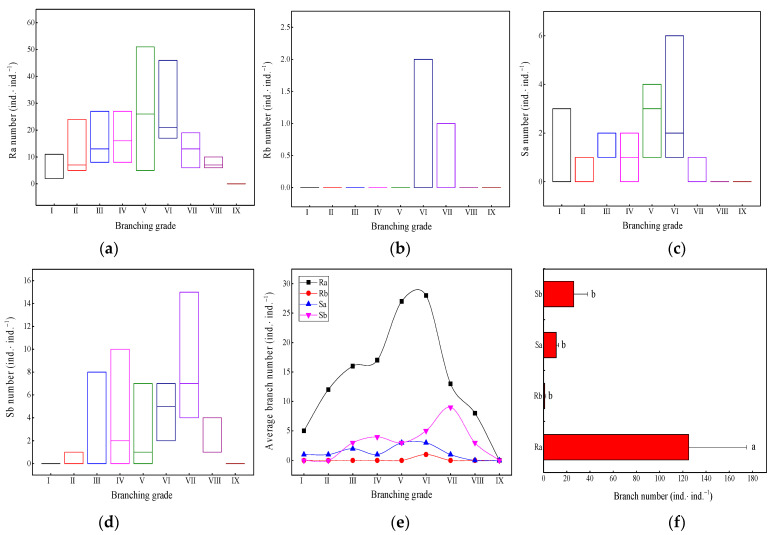
Quantitative changes in different branches. (**a**) Ra number, rhizome branch number; (**b**) Rb number, rhizome bud branch number; (**c**) Sa number, standing bamboo branch number; (**d**) Sb number, shoot bud branch number; (**e**) average branch number; (**f**) the total number of different branching types. Different letters indicate significant differences (*p* < 0.05). Unit: ind.·ind.^−1^ is individual branch per individual ramet system.

Among the four branch types in the ramet system, except for the Rb (rhizome bud) branch, which only existed for 1–2 ind.·ind.^−1^ at branching grades VI and VII (Figure 3b), the Ra branch (Figure 3a), Sa branch (Figure 3c), and Sb branch (Figure 3d) at the intermediate branching grades in the ramet system had a large range of variation. The average number of Ra branches in the ramet system (Figure 3e) showed a significant “unimodal” change, with the largest amplitude of change reaching its peak at branching grades V and VI. Except for the significant increase in the average number of Sb branches at branching grade VII, the change in the average number of other branches was nonsignificant. In each branch type of the ramet system (Figure 3f), the number of Ra branches was significantly higher than that of the other branch types (*p <* 0.05). The number of underground branches (Ra and Rb) in the ramet system was as high as 126 ind.·ind.^−1^. The number of aboveground branches (Sa and Sb) was 37 ind.·ind.^−1^.

### 2.4. Branch Distribution of Ramet System

**Figure 4:**
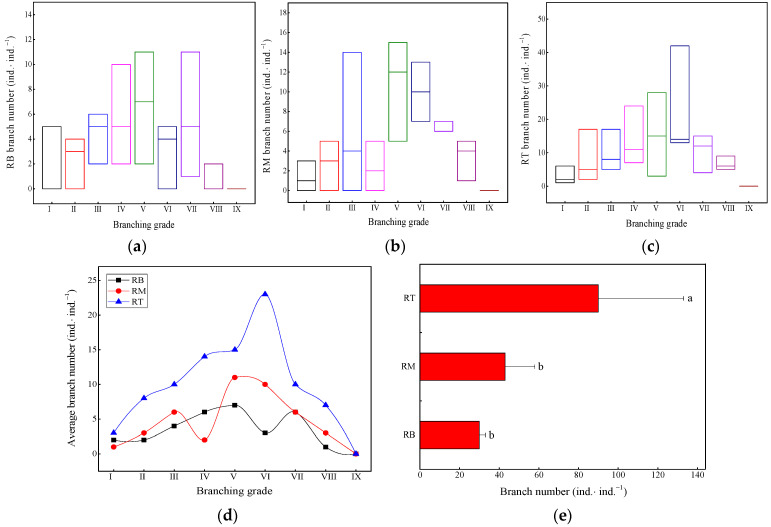
Quantitative changes in branch distribution. (**a**) RB branch number, rhizome base branch number; (**b**) RM branch number, rhizome middle branch number; (**c**) RT branch number, rhizome tip branch number; (**d**) average branch number; (**e**) differences in branch distribution. Different letters indicate significant differences (*p* < 0.05). Unit: ind.·ind.^−1^ is individual branch per individual ramet system.

The variation in the number of branches (Figure 4a–c) in the intermediate branching grades of the ramet system was relatively large. The branch distribution of the RT (Figure 4d) showed a significant “unimodal” change with increasing branching grades. The peak branch distribution of the RT, RM, and RB was generally located at branching grades V–VII (Figure 4d). The number of branches in the RT was the highest (Figure 4e), which was significantly higher than the distribution of branches in the other parts (*p <* 0.05). The number of branches in the RB was the lowest, and there was no significant difference from that of the RM (*p >* 0.05).

## 3. Discussion

### 3.1. Existence Form of Bamboo Ramet System

Asexual plants can form differently sized ramet systems containing different underground rhizome lengths and multiple ramets. Through the regulation of ramet system size, they strongly affect the effective utilization of their rhizosphere nutrients [19]. The ramet system also changes the position of ramets and the length of underground rhizomes to obtain the necessary nutrients [20], indicating the potential roles of the ramet system’s size and structure in adapting to habitats. This study found that in bamboo forests, as the branching grade increased, the average length of bamboo rhizomes, the number of lateral buds and Ra branches (highest in different branching types), and the distribution of RT branches (highest in different branching positions) showed a trend of first increasing and then decreasing, clearly presenting a structural feature of “larger in the middle and smaller at both ends”. The type of plant growth structure determines its functional role [21]; therefore, this structural type of the bamboo ramet system in the field inevitably affects the performance of related functions during the growth process.

In this study, the average rhizome length of the bamboo ramet system was the highest in branching grade VII, and the average number of dormant buds was significantly higher than in branching grade III. The average number of mortal buds was higher than that of dormant and germinated buds of branching grades I–V. The average number of germinated buds peaked at branching grade VI, indicating that the vitality of each branch and the lateral bud vitality of the underground rhizome gradually increased from the initial to the distal end of the ramet system. The lateral buds that grew at the distal end of the ramet system were mainly dormant buds, and those that grew at the last branching grade were all dormant buds; the number of germinated buds at the intermediate branching grade was relatively small, and the number of mortal buds was relatively high. Except for a handful of germinated branches, most lateral buds gradually aged and died, and the initial branching grade was dominated by dead buds. Research showed [22] that differences in the length of underground rhizomes in the ramet system significantly affect the energy required for resource transport and limit the intensity of clonal integration, especially because the resource transport capacity of aging underground rhizomes is weakened. The redistribution of resources between ramets is also significantly affected by the number of ramets [23], especially because the ability of aging ramets to redistribute resources is weakened. Therefore, to improve the “work efficiency” of bamboo ramet systems, removing the initial part of the system that gradually loses its vitality during the bamboo forest management process is necessary not only to increase the underground expansion space of the bamboo ramet system but also to maintain the high-vitality growth of the system.

### 3.2. Reduction and Expansion of Bamboo Ramet System

Most studies have focused on the expansion process of asexual plant populations and the impact of various human or environmental factors on their expansion [24], usually neglecting an important link in population growth dynamics: the reduction process of the ramet system. We found that the initial branches of the bamboo ramet system (mainly branching grades I–III) belonged to the aging branches. In intensively managed bamboo forests, aging underground ramet system rhizomes are mainly manually cleared during the forest land reclamation process, and those not cleared gradually die and rot. Thus, the morphological growth and branching quantity at the initial end are relatively small. As the aging-end branches are cleared or die annually, the branching grade of the ramet system gradually decreases.

The asexual expansion of plants is a complex and important process that affects their growth performance and how they acquire environmental resources [25]. The continuous branching of the ramet system indicates a continuous expansion of the population. We found that the intermediate positions of the bamboo ramet system (mainly including branching grades IV–VI) were mainly composed of Ra and Sa branches, and the branches near the distal end were mainly Sb, with a small amount of Rb branches. These results revealed that the main function of the lateral buds of the intermediate part of the underground rhizome is bud output. As the bamboo rhizomes at intermediate branching grades gradually develop and mature, the lateral buds growing on them continuously branch into new bamboo rhizomes and standings, contributing to the large number of branches at intermediate branching grades in the ramet system. Owing to the annual branching growth of the intermediate part of the ramet system, the branching grade of the ramet system gradually expands.

We also found that the length of the bamboo rhizomes, number of different types of bud banks, number of different types of branches, and number of branches at different branching positions in the intermediate grades of the bamboo ramet system varied substantially, which may have been due to the frequent reclamation of mulched bamboo forests and the large number of bamboo shoots being dug, resulting in rhizome breakage. Breaking rhizomes causes varying degrees of fragmentation in the ramet system. Disturbances are inevitable in the natural environment, and the greatest potential risk for clonal plants after disturbances is clonal fragmentation [26]. Dai et al. [27] showed that fragmentation negatively affected the growth performance of clonal plants. Therefore, in the process of bamboo forest management, improving the precision control of the manual management of bamboo ramet systems can avoid the fragmentation of bamboo ramet systems caused by disorderly human interference, thus reducing the impact on bamboo growth and forest productivity.

### 3.3. Renewing and Moving of Bamboo Ramet System

Bud banks are an important source of nutritional reproduction and expanded growth for clonal plants and play a crucial role in the stability and productivity of plant populations [28]. In this study, the final branching grade at the distal end (mainly including branching grades VI–IX) of the ramet system belonged to the newly formed end and was mainly composed of newly growing bamboo rhizomes. The lateral buds on them were not yet mature and did not have the function of branching into shoots or rhizome buds, explaining the small quantity of growth and branching characteristics of the distal branching grades. The main function of the newly formed ends of the bamboo ramet system is bud input. In perennial clonal plant populations, the metabolism and growth costs of buds are very low. Maintaining many dormant buds is an important growth strategy for plants, with the greatest benefit being that they can quickly respond to external environmental changes by producing many ramets [29]. Therefore, the bamboo ramet system relies on the continuous input of buds from the newly formed ends to maintain sustainable growth.

The underground rhizome lateral buds of clonal plants can grow upward to form new ramets or horizontally to form new underground rhizome branches, promoting the growth and expansion of plant populations [30]. Owing to the unique underground and aboveground branching and growth characteristics of bamboo ramet systems, bamboo has become a mobile plant. The initial end of the ramet system is constantly decreasing due to slow aging and death or manual clearance, and as the initial end decreases, the original ramet system continuously disintegrates. After disintegration, each independent ramet system continues to expand as it branches and grows; with the expansion of the ramet system, that is, the continuous growth of its branches and peripheral expansion, bamboo achieves continuous movement based on the basic unit structure of the ramet system. Owing to the continuous cycle of this process, the bamboo ramet system has become a truly “underground runner [31]”.

## 4. Materials and Methods

### 4.1. Investigation Location

The test site was located in Taihuyuan Town (29°56′–30°23′ N, 118°51′–119°72′ E), Lin’an District, Hangzhou City, Zhejiang Province, one of the “Hometowns of Bamboo” in China. This area has fertile soil, with an annual precipitation of 1250–1600 mm, an average annual temperature of 15.4 °C, and an annual sunshine duration of 1850–1950 h, which are suitable conditions for bamboo growth. This area is also one of the earliest areas in China in which bamboo forest mulching technology was applied.

Based on the current conditions, the investigation was conducted in a 6-year mulched bamboo forest with similar site conditions and management levels, and no signs of degradation in bamboo population growth. Soil samples were collected from different branching grades of the ramet system, and their properties are listed in Table 1. The bamboo forest adopted a continuous mulching model for 2 years and 1 year of rest, with cumulative mulching until the 6th year. The mulch material was wheat bran as the fermentation layer, with a thickness of 10–15 cm, and rice husk as the insulation layer, with a thickness of approximately 15 cm. The number of bamboo standings in the bamboo forest was maintained at 12,000–15,000 standings·ha^−1^, and only 1- to 4-year-old bamboo standings remained for cultivation. The diameter at the breast height of the bamboo forests was mainly distributed between 3.00 and 3.50 cm.

### 4.2. Data Investigation and Analysis

#### 4.2.1. Method of Tracking Bamboo Rhizomes through Bamboo Standings

**Figure 5:**
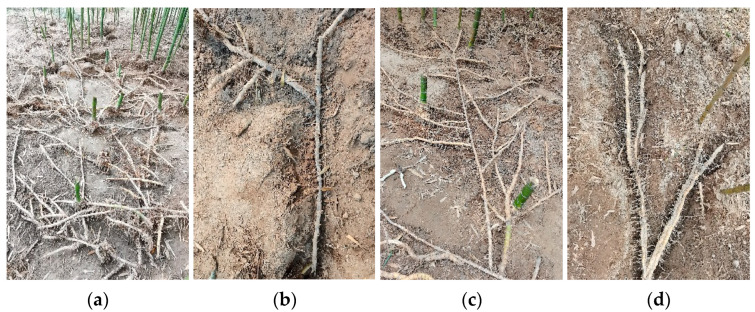
(**a**) Bamboo ramet system; (**b**) initial branches, black and withered; (**c**) intermediate branches, yellow and strong; (**d**) distal branches, white and tender.

In the bamboo forest, one bamboo standing with an average diameter at breast height was randomly selected and cut from a distance of 20–30 cm from the ground (three replicates). The bamboo standing was dug up such that all bamboo rhizomes and standings (all standings were sawn off at a distance of 20–30 cm from the ground for statistical purposes) connected to it were excavated. If the selected bamboo standing was old bamboo, all branches in the direction of the incoming rhizome were excavated first; if the bamboo standing was new bamboo, all branches in the direction of the outgoing rhizome were excavated first. We attempted not to break all of the connected branches. Finally, the bamboo roots were cut off, the soil was cleaned, and the interconnected veins of the ramet system were made clearly visible (Figure 5).

#### 4.2.2. Investigation of Bamboo Rhizome Morphology and Bud Bank

The length and diameter of each bamboo rhizome were measured according to the branching grade of the ramet system. The number of rhizome internodes (rhizome internode length = bamboo rhizome length/rhizome internode number) was recorded. The total number of buds comprised the number of lateral buds of each bamboo rhizome that germinated into bamboo rhizomes, rhizome buds, bamboo standings, and shoot buds, and the number of dormant and mortal buds that grew.

#### 4.2.3. Branching Investigation of the Ramet System

The branching and growth of bamboo ramet systems have a certain directionality. The lateral buds growing on the underground rhizome always alternate along the direction of bamboo rhizome growth. If the lateral buds germinate into bamboo rhizomes, they grow horizontally underground. If the lateral buds germinate into bamboo shoots, they grow vertically aboveground. Thus, starting from the initial end of the ramet system to the distal end, we performed stepwise marking starting from branching grade I. All branches grown from the previous grade were recorded as the next grade. The different branches of each grade were marked sequentially along the direction of lateral bud growth, recording the branch type (bamboo rhizome (Ra), rhizome bud (Rb), bamboo standing (Sa), shoot bud (Sb)), and the number of attached rhizome internodes. The bamboo rhizomes were divided into three equal parts according to the number of rhizome internodes: rhizome base (RB), middle (RM), and tip (RT). Distribution patterns of the branches were calculated separately.

#### 4.2.4. Data Analysis

The mean and standard deviation were calculated using Microsoft Excel 2016 (Microsoft Corporation, Redmond, WA, USA). One-way random block analysis of variance was performed using SPSS version 19.0 (IBM Corp., Armonk, NY, USA), and the Student–Newman–Keuls test (i.e., q test) was used for multiple comparisons (drawn in Origin 2016, OriginLab Corporation, Northampton, MA, USA).

## 5. Conclusions

Bamboo exists as a ramet system in the field. In mulched bamboo forests, the intermediate branching grades of the ramet system have long bamboo rhizomes, many lateral buds, and many branches, and the initial and distal branching grades have short bamboo rhizomes and a small number of lateral buds and a few branches. The initial end of the ramet system gradually ages and decreases, causing the disintegration of the ramet system; the lateral buds at the intermediate branching grades continuously branch, promoting the expansion of the ramet system; and the distal end of the ramet system continuously inputs many dormant buds into the ramet system, achieving a renewal of the ramet system. Bamboo populations have successfully expanded into the surrounding habitats through continuous reduction, disintegration, expansion, and movement of the ramet system. The higher the level of artificial management, the greater the disturbance to bamboo forests. Thus, implementing precise regulation of the bamboo ramet system to avoid artificial fragmentation and improve the “work efficiency” of the system is necessary.

## Figures and Tables

**Table 1:** Basic properties of bamboo forest soil.

Branching Grade	pH	Organic Matter (g·kg^−1^)	Hydrolyzable N (mg·kg^−1^)	Available P (g·kg^−1^)	Available K (mg·kg^−1^)
Ⅰ	4.25 ± 0.44 a	29.09 ± 14.79 a	158.00 ± 27.22 a	148.90 ± 76.05 a	85.13 ± 18.47 a
Ⅱ	4.29 ± 0.22 a	38.67 ± 16.80 a	161.67 ± 23.54 a	151.90 ± 48.74 a	120.67 ± 17.90 a
Ⅲ	4.28 ± 0.18 a	30.10 ± 7.24 a	142.00± 11.00 a	114.00 ± 18.19 a	97.73 ± 20.82 a
Ⅳ	4.31 ± 0.28 a	29.85 ± 9.31 a	150.33 ± 21.50 a	105.37 ± 45.88 a	99.67 ± 15.04 a
Ⅴ	4.31 ± 0.24 a	27.62 ± 3.06 a	142.67 ± 8.08 a	126.07 ± 67.45 a	104.93 ± 22.23 a
Ⅵ	4.43 ± 0.24 a	40.53 ± 23.48 a	171.67 ± 35.53 a	124.67 ± 25.48 a	131.67 ± 24.38 a
Ⅶ	4.43 ± 0.20 a	29.07 ± 12.01 a	155.33 ± 3.79 a	136.20 ± 40.99 a	122.23 ± 34.26 a
Ⅷ	4.45 ± 0.29 a	34.93 ± 21.15 a	153.00 ± 19.47 a	126.90 ± 34.52 a	109.67 ± 19.55 a
Ⅸ	4.65 ± 0.16 a	26.85 ± 12.09 a	157.50 ± 9.19 a	146.00 ± 52.33 a	112.75 ± 22.98 a

Notes: Same letters indicate no significant differences (*p* > 0.05).

## Data Availability

Data are contained within the article.
